# Aichi Virus in Sewage and Surface Water, the Netherlands

**DOI:** 10.3201/eid1908.130312

**Published:** 2013-08

**Authors:** Willemijn J. Lodder, Saskia A. Rutjes, Katsuhisa Takumi, Ana Maria de Roda Husman

**Affiliations:** National Institute of Public Health and the Environment (RIVM), Bilthoven, the Netherlands (W.J. Lodder, S.A. Rutjes, K. Takumi, A.M. de Roda Husman);; Utrecht University, Utrecht, the Netherlands (A.M. de Roda Husman).

**Keywords:** Environmental, surveillance, Kobuvirus, Aichivirus, Aichi virus, sewage, surface water, 3C, VP1, PCR, viruses, the Netherlands, gastroenteritis, enteric infections

## Abstract

Detection of Aichi virus in humans was initially reported in Japan in 1989. To establish a timeline for the prevalence of Aichi virus infection among humans in the Netherlands, we conducted molecular analysis of archival water samples from 1987–2000 and 2009–2012. Aichi virus RNA was detected in 100% (8/8) of sewage samples and 100% (7/7) of surface water samples collected during 1987–2000 and 100% (8/8) of sewage samples and 71% (5/7) of surface water samples collected during 2009–2012. Several genotype A and B Aichi virus lineages were observed over the 25-year period studied, but the time course of viral genetic diversity showed recent expansion of the genotype B population over genotype A. Our results show that Aichi virus has been circulating among the human population in the Netherlands since before its initial detection in humans was reported and that genotype B now predominates in this country.

Gastroenteritis is a common waterborne disease in humans of all ages worldwide. Children and the elderly are most severely affected, especially in low-income countries (*1*). A number of viral etiologic agents, such as picornaviruses, caliciviruses, rotaviruses, human adenoviruses, and astroviruses, have been identified in the past few decades. However, a diagnostic gap remains in samples for which no causative agent is determined. It has been suggested that other picornaviruses may be involved (*2*).

Aichi viruses (species *Aichivirus*, genus *Kobuvirus*, family *Picornaviridae*) are small, nonenveloped viruses with a single-stranded, positive-sense RNA genome. Aichi virus in humans was reported in 1989 in Japan from a sample collected during an oyster-associated gastroenteritis outbreak (*3*); the complete nucleotide sequence of an Aichi virus was described in 1998 (*4*). Clinical signs and symptoms of Aichi virus infection include diarrhea, abdominal pain, nausea, vomiting, and fever, reflecting gastroenteritis (*3*,*5*). Aichi virus has been found at low incidence in patients with gastroenteritis in several regions around the world, including South America (*6*), Asia (*7*,*8*), Europe (*6*,*9*–*12*), and Africa (*13*). Serologic studies indicate that up to 90% of the human population has been exposed to Aichi viruses by the age of 40 years (*14*). However, the epidemiology of gastroenteritis caused by Aichi virus is, to a large extent, unknown.

Aichi viruses have mainly been detected by PCR targeting the 3CD junction of the virus genome (*15*). The 3CD junction region has been described as conserved, and the viral protein (VP) 1 region is more genetically diverse (*4*,*6*,*9*,*16*). VP1 sequence typing is standard for the classification of picornaviruses (*17*), but analysis of the 3CD region has been used to divide Aichi viruses into 3 genotypes: A, B, and C (*9*,*15*).

Aichi viruses excreted with human feces contaminate surface waters directly or after discharge of treated or untreated sewage (*18*). Humans could be exposed to these viruses in surface waters used for the production of drinking water (after insufficient treatment) or for recreational purposes and after consumption of raw shellfish cultivated in contaminated surface waters. One indication that Aichi viruses may be transmitted by the fecal–oral route is the detection of these viruses in sewage samples in Tunisia (*19*), in surface waters in Venezuela (*20*), and in sewage and river waters in Japan (*21*). Some of these studies demonstrated a high Aichi virus prevalence in water samples. Viruses in sewage are thought to reflect the viruses circulating in the human population, originating from asymptomatic and symptomatic persons (*22*). Hence, environmental surveillance studies are extremely useful to determine the circulation of viruses in the human population (*22*,*23*) and to obtain sequence information of the circulating strains.

To establish a timeline for the emergence of Aichi viruses among the human population in the Netherlands, archival sewage and surface waters sampled over a >25-year period were subjected to molecular analysis targeting VP1 and the 3C region of the Aichi virus genome. The detected viruses were typed by sequence analysis to determine genetic variability. These environmental Aichi virus strains were subsequently compared with strains previously isolated from clinical materials and environmental samples worldwide. The possible emergence of the Aichi virus infections in humans was inferred by analyzing the population dynamics of these Aichi viruses.

## Material and Methods

### Samples

Depending on water type and the pressure during the membrane filtration, different volumes of water were concentrated by using a conventional filter adsorption-elution method. The resulting eluates were further concentrated by using an ultrafiltration method as described (*24*). The remaining samples were stored at −70°C or −30°C. 

Fifteen archival concentrates from these samples, originating from 1987–2000, were randomly selected for analysis. Of these samples, 8 were raw sewage samples and 7 were surface water samples. The time of storage of the concentrates did not influence the results, as a previous study also found (*25*). 

Fifteen additional archival samples, originating from 2009–2012, also were selected. Of these, 8 samples originated from raw sewage and 7 samples from surface waters (*18*,*26*); of the sewage samples, 4 were tested directly, without sample concentration. 

An Aichi virus–positive control, a culture supernatant of the Japanese isolate A846/88, was kindly provided by Erwin Duizer (Laboratory for Infectious Diseases and Perinatal Screening, National Institute for Public Health and the Environment [RIVM], Bilthoven, the Netherlands).

### RNA Extraction

Genomic material was isolated from 12.5-μL and 125-μL volumes of the water concentrates, corresponding to 26 mL–2,000 mL of the original surface water and 0.5 mL–176 mL of original sewage, depending on the concentration factor obtained by filtration. The NucliSENS miniMAG Nucleic Acid Isolation Kit (bioMérieux, Zaltbommel, the Netherlands) was used as described (*24*). 

For 4 raw sewage samples collected in 2010 and 2011, RNA was extracted directly from 1 mL and 5 mL of sewage. Nucleic acids were eluted from the silica in 100-μL elution buffer containing RNase inhibitor (Promega, Leiden, the Netherlands), and the eluate was further purified and concentrated by using the RNeasy MinElute Cleanup Kit (QIAGEN, Hilden, Germany). The extracted RNA was used directly in the reverse transcription reaction or stored at −70°C until use.

### cDNA Synthesis

cDNA was synthesized by using random hexamers. In brief, for each water concentrate, 5 μL of undiluted RNA and a sample of 10× diluted RNA were tested; in addition, a sample of 100× diluted RNA from each 125-μL water concentrate was tested. These volumes corresponded to 170 µL–140 mL of surface water and 3.4 µL–12 mL of sewage, depending on the extraction volume and the dilution factor. The RNA was added to 1.5 μg of random hexamers (Roche, Almere, the Netherlands) and the mixtures were heated at 70°C for 5 min and then chilled on ice for 5 min. Subsequently, 1X First Strand Buffer (Invitrogen, Leek, the Netherlands), 0.5 mmol/L dNTP (Roche), 2.5 mmol/L DTT (Roche), 0.2 U RNase inhibitor (Promega), and 5 U Superscript II (Invitrogen) were added at room temperature, resulting in a final volume of 20 μL. The mixture was incubated in a thermal incubator at 42°C for 60 min, heated at 95°C for 5 min, and then chilled on ice for 5 min. The synthesized cDNA was used directly in a PCR reaction or stored at −70°C until use.

### Nested PCR

#### VP1

For Aichi virus detection and typing, cDNA samples were amplified by a nested PCR using primers developed in this study to target the VP3 and VP1 regions (Table 1). In brief, an aliquot of 5 μL of synthesized cDNA was added to 45 μL of the first-round PCR reaction mixture containing 1X PCR reaction buffer with MgCl_2_ (Roche), 0.2 mmol/L dNTP (Roche), 1 μmol/L each primer (F1 and R1), and 2.5 U of Taq DNA Polymerase (Roche). The PCR protocol was as follows: a denaturation and activation step at 94°C for 5 min, followed by 35 cycles of 94°C for 30 s, 60°C for 30 s, and 72°C for 60 s. A 1-μL volume from the first-round PCR was used as a template for the second-round PCR mixture, containing 1X PCR reaction buffer with MgCl_2_ (Roche), 0.3 mmol/L dNTP (Roche), 0.1 μmol/L each primer (primer combinations F2/R2 and F3/R2 were used), and 2.5 U of FastStart Taq DNA Polymerase (Roche). The PCR protocol was as follows: a denaturation and activation step at 95°C for 5 min, followed by 40 cycles of 95°C for 20 s, 60°C for 30 s, and 72°C for 40 s. The second-round PCR products were separated on 2% agarose gels and visualized by ultraviolet illumination after staining with SYBR Gold Nucleic Acid Gel Stain (Molecular Probes, Leiden, the Netherlands) to identify positive samples. DNA fragments of 530 bp were amplified for primer pair F2/R2 and fragments of 264 bp for primer pair F3/R2. Positive second-round PCR products were purified by using a QIAquick PCR Purification Kit (QIAGEN), according to the manufacturer’s instructions. All purified PCR products were stored at −20°C until further use.

**Table 1:** Oligonucleotide sequences of the VP1 and VP3 primers developed and used for study of Aichi virus in sewage and surface water, the Netherlands*

Primer	Sequence, 5′→3′†	Nucleotide location‡	PCR
AiV-VP3-F1	CACACCGCCCCTGCGTCRGCCCTCGT	2912–2937	First-round
AiV-VP1-F2	CTCGATGCRCCMCAAGACACCGG	3023–3045	Nested
AiV-VP1-F3	GTGCTTCACRTACATCGCYGCGG	3289–3311	Nested
AiV-VP1-R2	CCTGACCAGTCCTCCCAWCCGAAGTA	3552–3527	Nested
AiV-VP1-R1	GAGAGCTGGAAGTCRAAGGG	3651–3632	First-round

*VP, viral protein; AiV, Aichi virus; F, forward; R, reverse. †R indicates A or G; M indicates A or C; Y indicates C or T; W indicates A or T. ‡Compared with Aichi virus reference strain (GenBank accession no. AB010145).

#### 3C Region

For Aichi virus detection and typing, cDNA samples were amplified by a nested PCR using primers targeting the 3C region as described (*6*). PCR mixtures and protocol were as described for the VP1 PCR and amplified by using the same cycling parameters, except that the annealing step in the second-round PCR was performed at 55°C. For the first-round PCR, DNA fragments of 313 bp were amplified; for the second-round PCR, fragments of 180 bp. Positive PCR products were purified and stored at −20°C until further use.

### Cloning and Sequencing

The purified PCR products were cloned into a pCRII-TOPO Vector (Invitrogen), according to the manufacturer’s instructions; the construct was subsequently transformed into JM109 competent cells. Approximately 5–7 clones were randomly selected per purified PCR product and were checked by using M13 primers supplied by the manufacturer (Invitrogen). Up to 6 positive clones from each sample were randomly selected and subjected to sequence analysis of both strands with M13 primers by using a BigDye Terminator Cycle Sequencing Ready Reaction Kit (Applied Biosystems, Foster City, CA, USA).

### Phylogenetic Analysis

The obtained 3C and VP1 sequences were edited with BioNumerics software version 6.6 (Applied Maths, Kortrijk, Belgium) and compared with all available Aichi virus sequences from GenBank. Multiple DNA sequences from each genotype were aligned by using MAFFT version 6.847b (*27*). We estimated phylogenies of the dated samples using a Bayesian Markov chain Monte Carlo method implemented in the Bayesian evolutionary analysis by sampling trees (BEAST version 1.7.4 [*28*]) and estimated coalescent effective population sizes using skyline plots (*29*). Skyline plots represent a nonparametric flexible method based on coalescent theory; the method was used to reconstruct changes in population sizes through time. The Hasegawa-Kishino-Yano model of DNA evolution with a uniform mutation rate across branches (strict clock) was used with default priors. Simulations were run for 30 million updates after discarding burn-in. The resulting tree was summarized by using TreeAnnotator version 1.7.4 (*28*), and the maximum-clade credibility tree was visualized and edited with FigTree software version 1.3.1 (http://tree.bio.ed.ac.uk/software/figtree/).

## Results

We found Aichi viruses were present in sewage and surface water samples originating from both sampling periods, 1987–2000 and 2009–2012 (15 samples from each period). Aichi virus RNA was detected in 93% (28/30) and 83% (25/30) of water samples by testing that targeted the 3C and VP1 regions, respectively. Aichi virus RNA was detected by both detection methods in all 16 sewage samples from both sampling periods and in 12 (86%) of 14 and 9 (64%) of 14 surface water samples for the 3C region and the VP1 region, respectively (Table 2).

**Table 2:** Sample characteristics and summarized results per genomic region of sewage and surface water samples collected during 1987–2000 and 2009–2012 and tested for Aichi virus, the Netherlands*

Sample no.	Sampling month	Sample type	Genotype†	
			3C	VP1
1987-49	July	Sewage	B	B
1987-56	August	Surface water	B	B
1987-75	September	Surface water	B	B
1989-33	April	Sewage	A, B	B
1991-29	April	Sewage	B	A, B
1994-10	February	Surface water	A	A
1995-44	July	Surface water	B	ND
1997-27	May	Surface water	A	ND
1997-31	June	Sewage	A	A
1997-39	June	Sewage	A	B
1998-20	February	Surface water	A, B	A
1998-56	May	Sewage	‡	A
1998-62	May	Sewage	A, B	A
1999-46	April	Sewage	CK	B
2000-12	February	Surface water	A	ND
2009-011	January	Sewage	B	B
2009-074	May	Surface water	B	B
2009-075	May	Surface water	B	B
2009-064	April	Surface water	B	B
2010-007	January	Surface water	B	B
2010-033	March	Surface water	B	B
2010-210	September	Sewage	B	B
2010-216	October	Sewage	B	B
2011-024	February	Sewage	CK	A
2011-129	May	Sewage	B	B
2011-221	June	Sewage	B	B
2011-579	September	Sewage	A	B
2011-331	August	Sewage	B, CK	B
2012-063	April	Surface water	ND	ND
2012-195	June	Surface water	ND	ND

*VP, viral protein; ND, not detected; CK, canine kobuvirus. †Result per genomic region. ‡Not clustering with the known genotypes A, B, or C.

**Figure 1:**
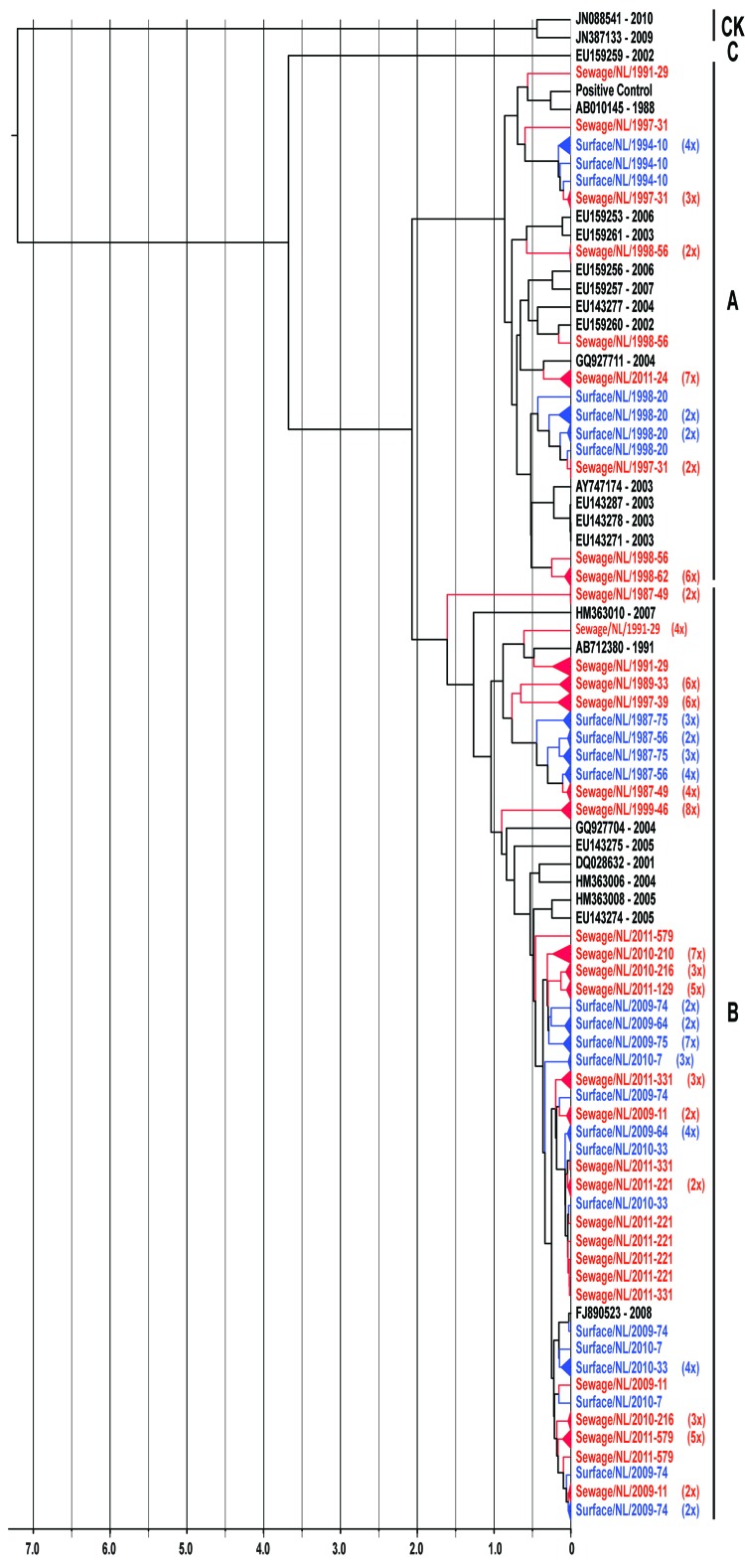
Maximum-clade credibility tree showing the phylogenetic relationships between Aichi virus isolates from the Netherlands and other locations, based on a multiple alignment of nucleotide sequences (481-nt) of the viral protein (VP) 1 region. The rooted tree was generated by the Bayesian Markov chain Monte Carlo method in BEAST (*28*), using CK as an outgroup, visualized in FigTree (http://tree.bio.ed.ac.uk/software/figtree/), and plotted on a temporal *y*-axis scale in units of 1,000 years. Aichi virus strains from the Netherlands isolated from sewage (red) and surface waters (blue) are indicated; reference strains (black) are shown with GenBank accession numbers. Clusters of sequences of the same sample are represented by triangles (a collapsed branch), and the number of isolates in each triangle is shown in parentheses. Aichi virus genotypes are shown on the right (A, B, and C). CK, canine kobuviruses.

**Figure 2:**
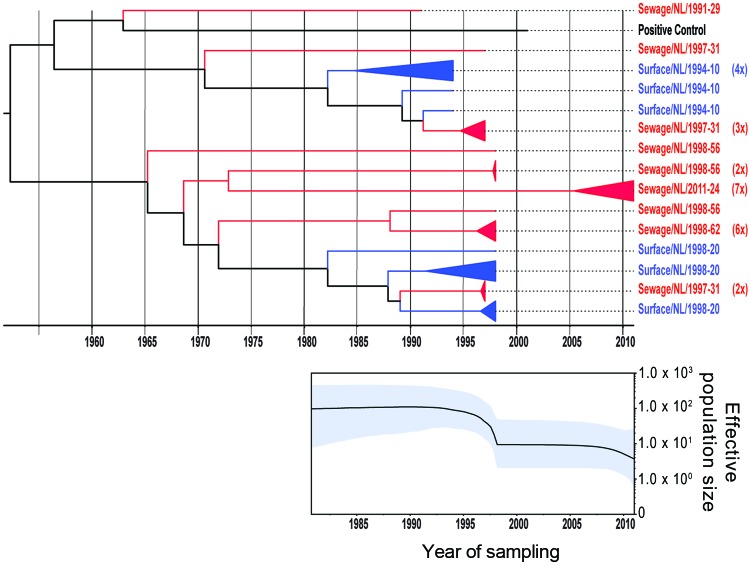
Phylogenetic relationships and genetic diversity over time for 37 sequences of Aichi virus genotype A strains collected in the Netherlands. A) Maximum-clade credibility tree was generated by the Bayesian Markov chain Monte Carlo method in BEAST (*28*), based on a multiple alignment of nucleotide sequences (481-nt) of the viral protein 1 region. The tree is rooted to the most recent common ancestor, visualized in FigTree (http://tree.bio.ed.ac.uk/software/figtree/), and plotted on a temporal *y*-axis scale using the sampling dates. Aichi virus strains from the Netherlands isolated from sewage (red) and surface waters (blue) are indicated. Clusters of sequences of the same sample are represented by triangles (a collapsed branch), and the number of isolates in each triangle is shown in parentheses. B) Bayesian skyline plot obtained by analyzing different Aichi virus sequences sampled at different times. The results are a relative measure for genetic diversity through time. The line represents the median, and the shaded area represents the 95% highest posterior density of the number of isolates.

**Figure 3:**
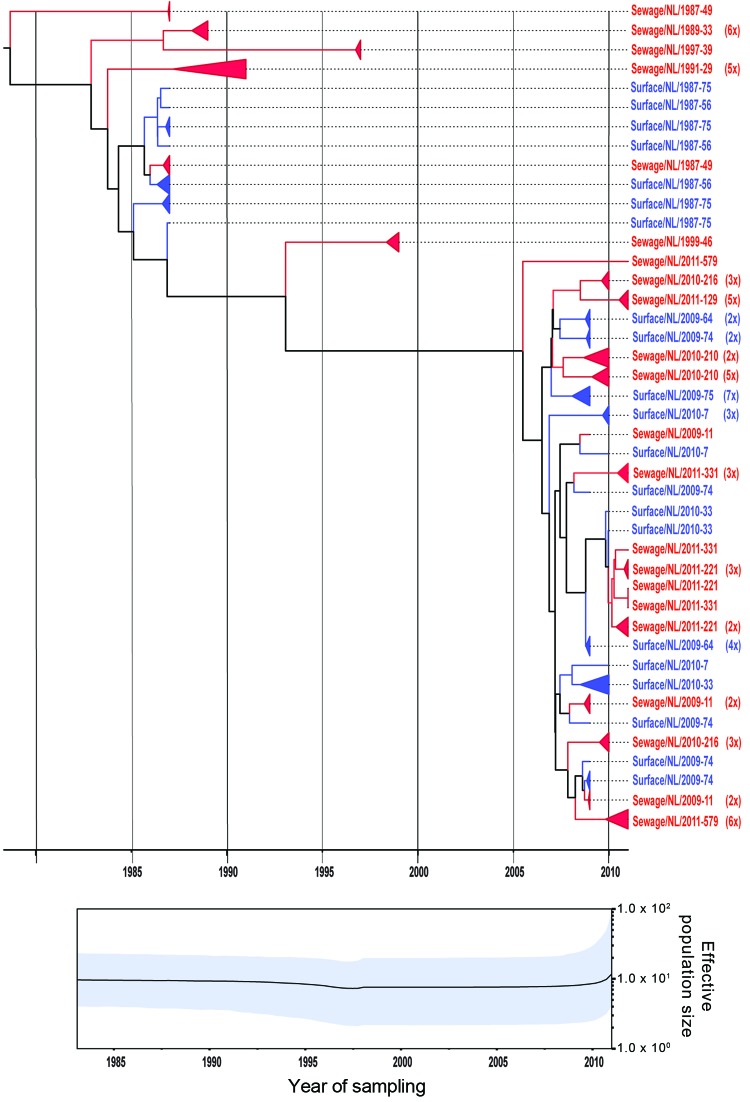
Phylogenetic relationships and genetic diversity over time for 166 sequences of Aichi virus genotype B strains collected in the Netherlands. A) Maximum-clade credibility tree was generated by the Bayesian Markov chain Monte Carlo method in BEAST (*28*), based on a multiple alignment of nucleotide sequences (481-nt) of the viral protein 1 region. The tree is rooted to the most recent common ancestor, visualized in FigTree (http://tree.bio.ed.ac.uk/software/figtree/), and plotted on a temporal *y*-axis scale using the sampling dates. Aichi virus strains from the Netherlands isolated from sewage (red) and surface waters (blue) are indicated. Clusters of sequences of the same sample are represented by triangles (a collapsed branch), and the number of isolates in each triangle is shown in parentheses. B) Bayesian skyline plot obtained by analyzing different Aichi virus sequences sampled at different times. The results are a relative measure for genetic diversity through time. The line represents the median, and the shaded area represents the 95% highest posterior density of the number of isolates.

The 2 primer pairs, F2/R2 and F3/R2, used in the VP1 second-round PCR showed similar results. Because of the larger product of the F2/R2 primer pair (530 bp), PCR products obtained with this primer pair were further used for cloning and sequence analysis. Viral population dynamics was estimated over time by using Bayesian coalescent analysis of the VP1 nucleotide sequence alignment of the isolates from the Netherlands and GenBank reference strains. The phylogenetic tree in Figure 1 shows that the 151 sequences obtained from the strains isolated from the Netherlands were in several different clusters and clustered between the limited available GenBank sequences obtained from other countries. To explore the possible expansion of Aichi virus lineages in the Netherlands in the sampled period, the genetic variability of the genotype A and B Aichi viruses was analyzed separately (Figure 2, panel A; Figure 3, panel A). The phylogeny of genotype A Aichi viruses showed predominantly strains from samples taken early in the sample period; only 1 genotype A was found in a location sampled in 2011 (Figure 1; Figure 2, panel A; Table 2). Genotype B showed 2 distinct clusters that resulted in a ladder-like structure suggesting a continual replacement of lineages through time (Figure 1; Figure 3, panel A). Translation into amino acid sequences of VP1 showed a high similarity (<4% difference) between the genotype A strains and genotype B strains, but 2 separate clusters of genotype A and B were seen (data not shown).

A Bayesian skyline plot model was used to reconstruct a time course of viral genetic diversity (*30*). Although the dataset is limited (36 clones from 7 samples), a constant diversity was seen in genotype A Aichi virus strains detected until the 1990s, followed by an apparent drop in genotype A detection (Figure 2, panel B). A constant diversity was also seen in genotype B strains (Figure 3, panel B). Overall, genotype B Aichi viruses have been more prevalent in the Netherlands in the past decade than have genotype A Aichi viruses. 

**Figure 4:**
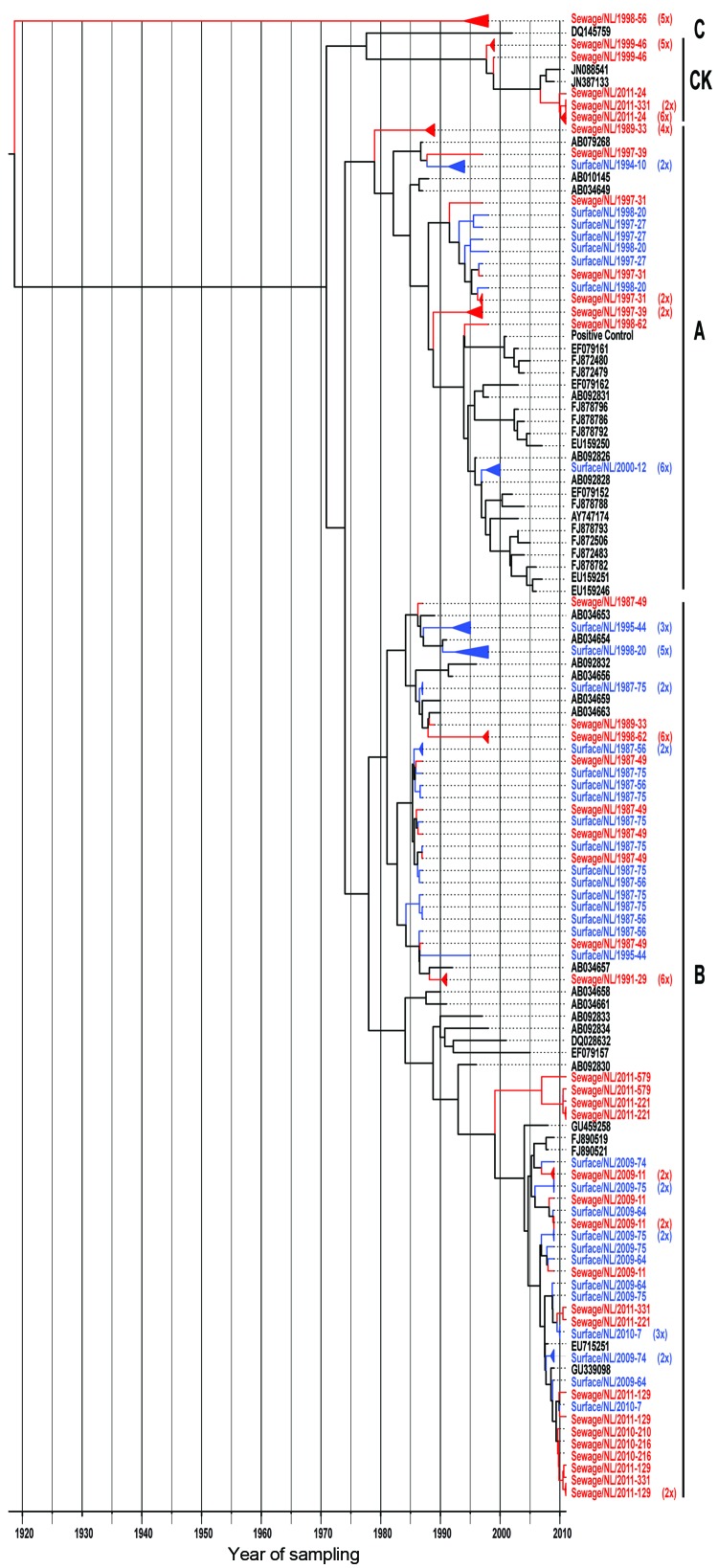
Maximum-clade credibility tree showing the phylogenetic relationships between Aichi virus isolates from the Netherlands and other locations, based on a multiple alignment of nucleotide sequences (139-nt) of the 3C region. The tree was generated by the Bayesian Markov chain Monte Carlo method in BEAST (*28*), rooted to the most recent common ancestor, visualized in FigTree (http://tree.bio.ed.ac.uk/software/figtree/), and plotted on a temporal *y*-axis scale using the sampling dates. Aichi virus strains from the Netherlands isolated from sewage (red) and surface waters (blue) are indicated; reference strains (black) are shown with GenBank accession numbers. Clusters of sequences of the same sample are represented by triangles (a collapsed branch), and the number of isolates in each triangle is shown between brackets. Aichi virus genotypes are shown on the right (A, B, and C). CK, canine kobuviruses.

After cloning of the 3C positive PCR products, phylogenetic analysis of the cloned sequences of the 3C region showed several different clusters within the known genotypes A and B (Figure 4). The phylogenetic tree in Figure 4 shows that the 127 sequences obtained from the environmental strains in this study were more divergent than the 3C sequences of Aichi viruses obtained from GenBank. No obvious differences were seen in the number of strains found in sewage or surface water. In the samples originating from 1987–2000, 3C sequences clustered with the known genotypes A and B and with canine kobuvirus strains. In contrast, for the samples originating from 2009–2011, sequences clustered only with genotype B and canine kobuvirus strains. The genotype B sequences obtained from both sampling periods showed 2 distinct clusters; the sequences of the first sampling period disappeared after 2009. Nevertheless, the amino acid sequences of the genotype A and B strains were very similar and did not show distinct clusters (data not shown). The phylogeny of the 3C sequences of genotype A and B Aichi viruses showed a ladder-like structure suggesting a continual replacement of lineages over time (Figure 4).

The sequence obtained from a sewage sample collected in 1998 (sewage/NL/1998-56) differed by up to ≈20% from the available Aichi virus sequences in GenBank (genotypes A, B, and C) (Figure 4). This high nucleotide divergence could suggest that this strain belongs to a new genotype of Aichi virus. Three sewage samples contained sequences that were highly similar (95%–96%) to recently discovered canine kobuviruses (GenBank accession nos. JN088541 and JN387133; Figure 4).

## Discussion

Reuter et al. (*14*) showed that the seroprevalence of Aichi virus in the human population worldwide is high. Up to 95% of persons 30–40 years of age have antibodies against Aichi virus, indicating a high rate of exposure to the viruses. By contrast, Aichi viruses are found at low incidence in clinical materials (*7*–*11*,*13*,*31*). More data are needed to gain better insight into the epidemiology and pathogenesis of Aichi viruses. Environmental surveillance of enteric viruses may give information on the possible circulation of Aichi viruses in the human population in the Netherlands, as well as their evolutionary dynamics. Therefore, we tested different water samples collected in the Netherlands over a period of 26 years (1987–2012) and found a high prevalence of Aichi viruses in samples from sewage and surface waters. We dected Aichi virus RNA in water samples from the Netherlands originating from 1987, which precedes description of Aichi virus in the literature, in fecal samples from patients affected in a 1989 oyster-related gastroenteritis outbreak in Japan (*3*).

Three previous studies have described the detection of Aichi viruses in environmental samples and found different prevalence levels. In Venezuela, 5 of 10 tested surface water samples contained Aichi virus RNA (*20*), but in Tunisia, only 15 of 250 (6%) tested raw and treated sewage samples contained Aichi virus RNA (*19*). Much higher prevalence was found in samples from Japan: raw sewage, 100% (12/12); treated sewage, 92% (11/12); and surface water, 60% (36/60) (*21*). Our study also found a high prevalence of Aichi viruses in water samples: 100% (14/14) of sewage and 85% (12/14) of surface water samples. Two surface water samples from 2012 tested negative for the presence of Aichi virus RNA, but this may have been explained by the origins: a large lake and a storage reservoir for the production of drinking water. These sources are differentfrom the other waters tested, which included large, relatively polluted rivers. To resolve possible transmission routes, Aichi viruses could be quantified in source waters for drinking water production and recreational waters by cell culture methods followed by quantitative microbial risk assessment to estimate public health risks from such exposures (*32*).

Several studies have compared the available Aichi virus sequences of the 3CD and VP1 regions and described the 3CD junction region as conserved and the VP1 region as more variable (*4*,*6*,*9*,*16*). Lukashev et al. (*33*) showed that the VP1 genome region, coding for structural proteins that express the antigenicity of the virus, is particularly suitable for distinguishing subtypes of Aichi viruses, whereas the sequence data of the more conserved 3CD junction region did not seem to provide sufficient sequence diversity for subtyping. The 3CD region, however, may be useful for the detection of a wider range of Aichi virus genotypes. The primers targeting the VP1 region used in this study proved to be useful for the detection of Aichivirus genotypes A and B. Sequence comparison with the limited sequence information of the other genotypes showed that our primers may have detected the Aichi virus genotype C less sensitively. In addition, the canine kobuviruses might not be detected by these VP1 primers; this may also be the case with the Aichi virus type found in sewage in 1998. More sequence information for the circulating Aichi virus strains is necessary to elucidate the considerations of a new genotype. Metagenomic studies, in both fecal and sewage samples (*34*,*35*), may facilitate the detection of new Aichi virus genotypes (or other genera of the kobuviruses), which also will facilitate the development of suitable primers for the detection of more Aichi virus genotypes.

Future research might focus on analysis of samples from gastroenteritis outbreaks for which no causative agent can be detected, using the 3C and VP1 primer sets described in this study. The resulting prevalence data could then be compared with the environmental surveillance data and to Aichi virus prevalence rates in fecal samples from persons of different ages with and without clinical illness. The results could elucidate the role of Aichi viruses in disease development and the severity of symptoms. Moreover, the susceptibility of vulnerable groups to Aichi virus infection and disease should be determined because disease was recently detected in elderly hospitalized patients with diarrhea in Sweden (*10*).

In our study, PCR products were cloned before sequence analysis so that we could detect multiple Aichi virus strains in 1 sample, not just the predominant strain. This resulted in the finding of multiple strains in 5 of the analyzed samples: sewage/NL/1989-33; sewage/NL/1991-29; surface/NL/1998-20; sewage/NL/1998-62; and sewage/NL/2011-331. We found a divergent Aichi virus strain in a sewage sample collected in 1998 (sewage/NL/1998-56) by comparing the nucleotide sequences of the 3C region with the known Aichi virus types available in GenBank (Figure 4). A nucleotide difference of ≈20% from the available sequences of genotypes A, B, and C was observed, tentatively leading to the conclusion that this sequence might belong to a new genotype of Aichi virus. More sequence information is needed to substantiate this finding by isolating this Aichi virus strain and subsequently subjecting the virus to whole-genome sequencing, as was described for an Aichi virus isolated in Taiwan in 2010 (*36*). For 3 of the sewage water concentrates from our study, 3C sequences were detected that showed high similarities with the recently discovered canine kobuvirus (*37*,*38*) (Figure 4). Although canine kobuviruses could have ended up in the sewage by run-off, further studies should be performed to gain more information about possible zoonotic transmission of these viruses.

Comparing the Aichi virus nucleotide sequences from the 2 sampling periods, 1987–2000 and 2009–2011, demonstrated that mainly genotype A strains were detected in the samples collected during 1987–2000. Aichi virus genotype B was found in both periods, but the sequences seemed to cluster in 2 distinct branches, which showed a shift in predominance of genotype B Aichi viruses after 2005 (Figure 3). Further analysis of these sequences, using BEAST (*28*), showed evolution of these genotype B strains over time, which resulted in a ladder-like structure, suggesting a continual replacement of lineages over the study period.

In conclusion, our study showed a high prevalence of Aichi viruses in environmental water samples in the Netherlands over an extended period of time, with a possible increase in genetic diversity of genotype B Aichi viruses. The additional sequence data obtained in this study may aid in the analysis of the evolution of Aichi viruses. In addition, the results emphasize the need for further research to understand the relative importance of possible transmission routes of Aichi viruses; that knowledge could allow the implementation of effective control measures.
